# Recurrent Jarisch-Herxheimer reaction in a patient with Q fever pneumonia: a case report

**DOI:** 10.1186/1757-1626-1-360

**Published:** 2008-11-29

**Authors:** Stavros Aloizos, Stavros Gourgiotis, Konstantinos Oikonomou, Paraskevi Stakia

**Affiliations:** 1 Intensive Care Unit, 401 General Army Hospital of Athens, 14 Acheon Street, 15343, Ag. Paraskevi, Athens, Greece; 2 Second Surgical Department, 401 General Army Hospital of Athens, 41 Zakinthinou Street, 15669, Papagou, Athens, Greece; 3 Oncology Department, 401 General Army Hospital of Athens, 23 Filias Street, 15123 Marousi, Athens, Greece; 4 8 Pirgou Street, 16675, Glyfada, Athens, Greece

## Abstract

Q fever is a zoonotic disease caused by coxiella burnetii. The Jarisch-Herxheimer reaction (JHR) is associated with the antibiotic treatment of certain bacterial infections. We report a very rare case of a 36-year-old male with Q fever pneumonia that resulted in recurrent ARDS and presented the JHR during his treatment. The patient was admitted for treatment of community acquired pneumonia. He developed ARDS, was intubated and placed on mechanical ventilation. Doxycycline was empirically added to his antibiotic regiment. The patient presented an acute rise in temperature, tachycardia, tachypnea, hypoxia, hypotension and a temporary deterioration of his chest x-ray. The same 6-hour-long reaction which is known as JHR was presented another 3 times. Cultures were negative but antibodies against coxiella burnetii were positive. This case reminds us that any deterioration of a patient treated in the ICU should not be considered as a new septic episode and time should be allowed for the antibiotic regiments.

## Case presentation

**Figure 1:**
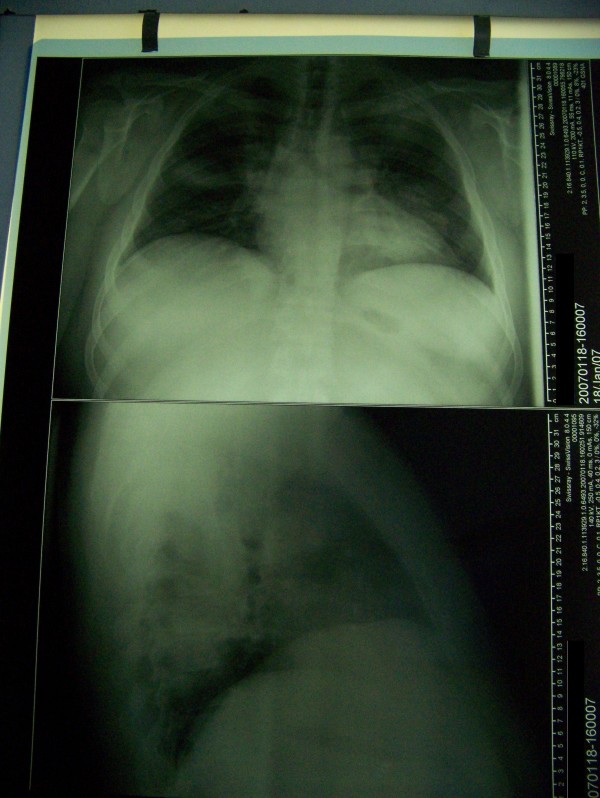
Chest x-ray shows bilateral pneumonia.

A 36-year-old man was presented with a 6-day history of producing cough, shortness of breath, pleuritic chest pain exaggerated on breathing, cold sweats, headache, joint and muscle pain. He was a countryside resident and had recently returned from a hunting trip. Chest x-ray revealed bilateral pneumonia (Figure 1). The initial treatment included 3^rd ^generation cephalosporin and cinolone for community acquired pneumonia.

**Figure 2:**
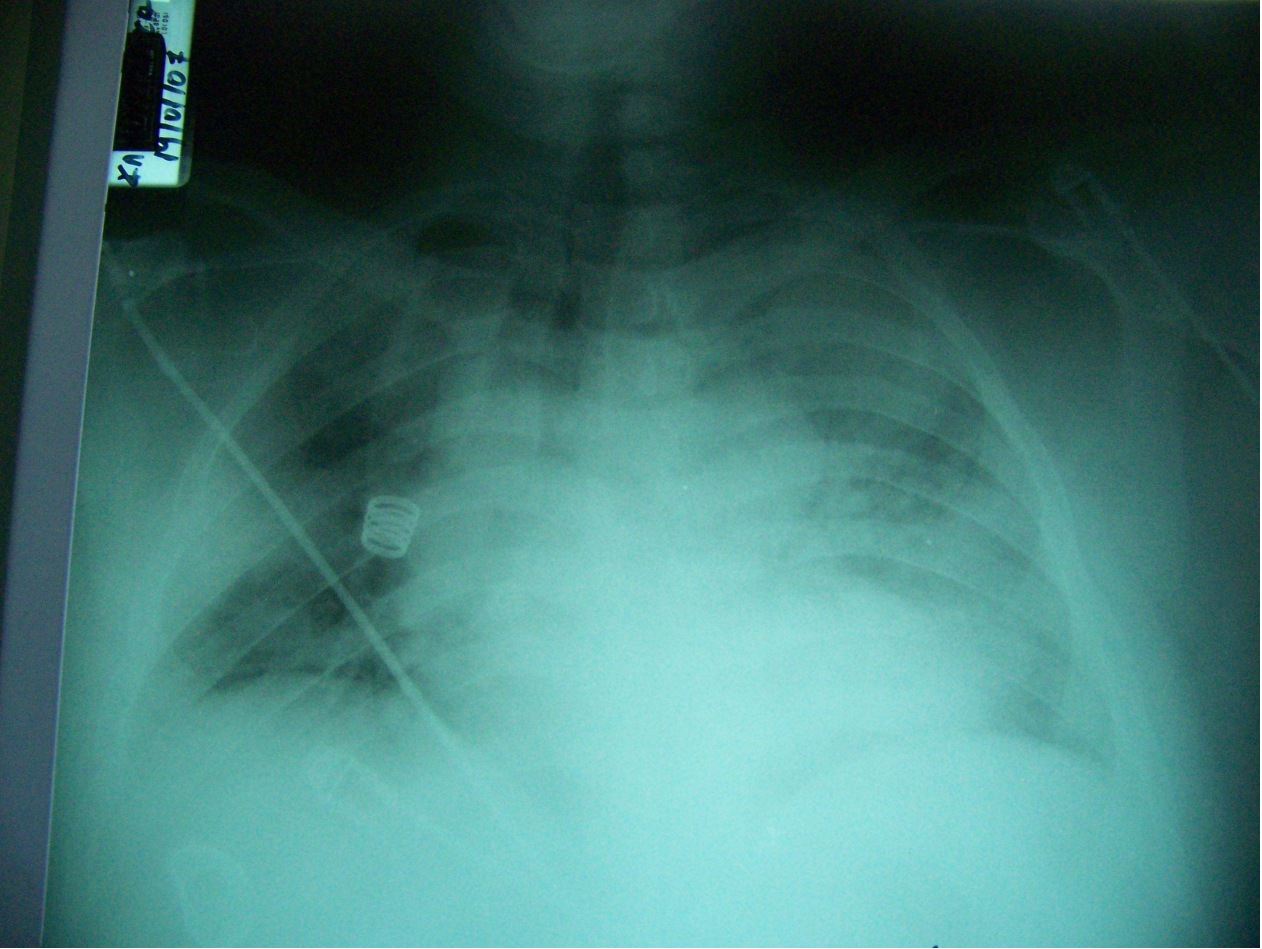
Chest x-ray demonstrates ARDS.

Hours after his admission, the patient fell into acute respiratory distress syndrome (ARDS) (Figure 2) and was transferred to the Intensive Care Unit (ICU) for further treatment. He was intubated and placed on mechanical ventilation which was extremely difficult (FiO_2_: 100%, high ventilator pressures and continuous infusion of muscle relaxants) for the first 48 hours. C-reactive protein (CRP) elevation was marked while patient's renal function had to be supported via dialysis.

**Figure 3:**
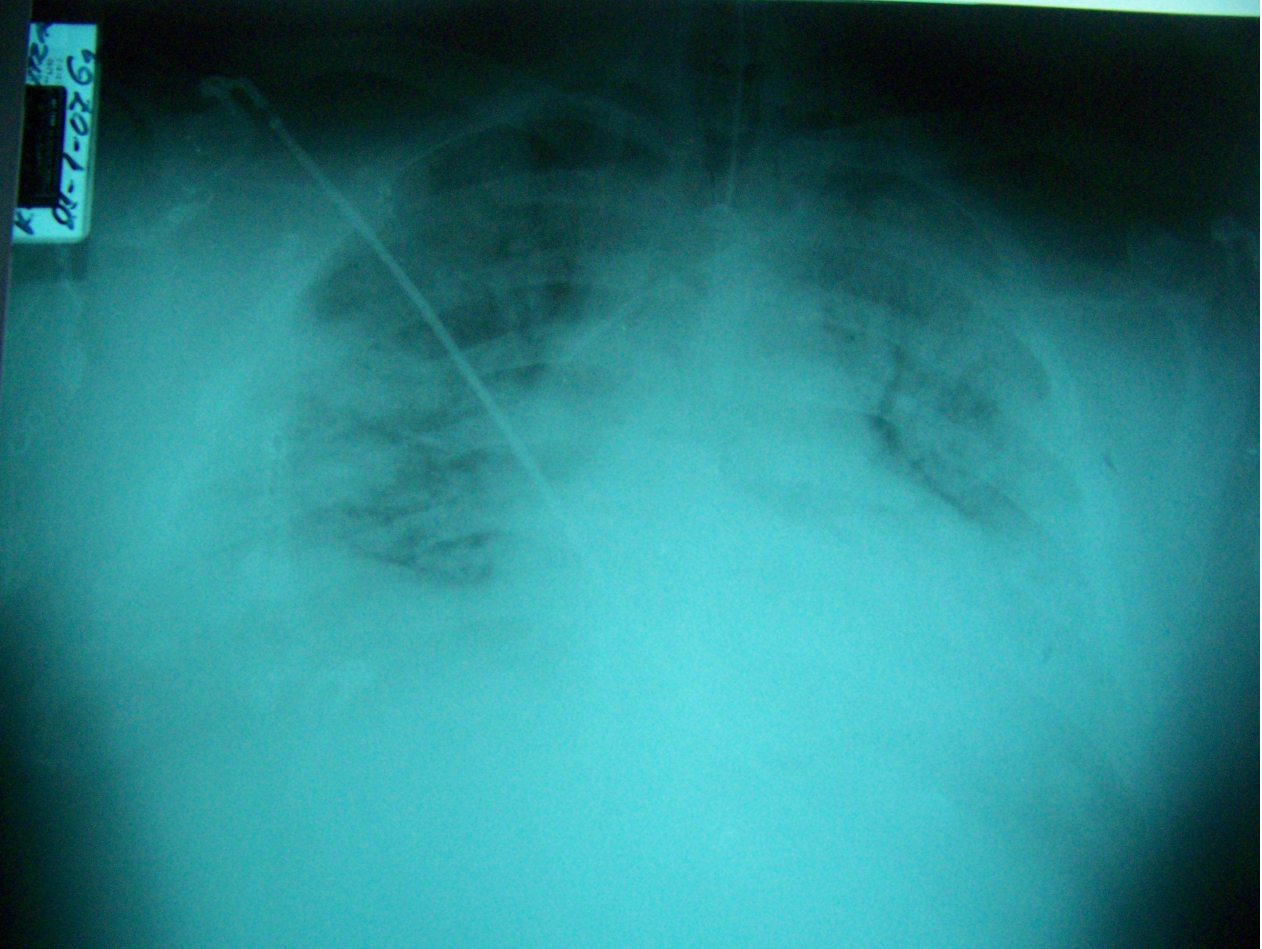
Chest x-ray of recurrent ARDS.

A few hours after the first doxycycline dose which was added empirically to the previous antibiotics, the patient presented an acute rise in temperature, tachycardia, tachypnea, hypoxia, hypotension and a temporary deterioration of his chest x-ray (Figure 3). The same 6-hour-long reaction which was later identified as Jarisch-Herxheimer reaction (JHR) was presented another 3 times. During these episodes, the patient had to be haemodynamically supported and transient high mixtures of oxygen according to the ARDSnet suggested treatment protocol which includes Tidal Volume (TV): 6–12 ml/kg, high level of PEEP & P plateau <30 mmHg and permissible hypercapnea [1]. Aggressive administration of insulin was also employed so as to maintain blood glucose levels between 70 and 90 mg/dl. The results of blood, urine and broncho-alveolar lavage (BAL) cultures were negative while the tests for antibodies against coxiella burnetii showed acute Q fever infection.

It should be noted that members of his family were subsequently tested and found also positive to Coxiella burnetii without any signs of clinical illness. Inflammatory cytokines were unfortunately not measured.

The patient was discharged 32 days after his initial admission.

## Discussion

Q fever is a zoonotic disease caused by coxiella burnetii, a species of bacteria with global distribution. It is now a notifiable disease in the United States but reporting is not required in many other countries. Because the disease is underreported, scientists cannot reliably assess how many cases of Q fever have actually occurred worldwide [2].

Cattle, sheep, and goats are the primary reservoirs of coxiella burnetii. The organisms are resistant to heat, drying and many common disinfectants which enables the bacteria to survive for long periods in the environment. Infection of humans usually occurs by inhalation of these organisms from air that contains airborne barnyard dust contaminated by dried placental material, birth fluids, and excreta of infected herd animals. Humans are often very susceptible to the disease and very few organisms may be required to cause infection.

50% of all people infected with coxiella burnetii show signs of clinical illness. Most cases begin with sudden onset of one or more of the following: high fevers (up to 40.5°C which usually lasts for 1 to 2 weeks), headache, general malaise, myalgia, confusion, sore throat, chills, sweats, non-productive cough, nausea, vomiting, diarrhea, abdominal and chest pain. Weight loss can occur and persist for some time [3]. 30–50% of patients with a symptomatic infection will develop pneumonia [4,5]. Most patients will recover to good health within several months without any treatment. Only 1%–2% of people with acute Q fever die of the disease [6].

The JHR occurs when large quantities of toxins are released into the body as bacteria (typically spirochetal bacteria, borellia or brucella) die due to antibiotic treatment. The proposed mechanism behind this is that the death of the bacteria and the subsequent release of endotoxins is faster that the rate the body can remove these toxins via its natural pathways (liver, kidneys) thus resulting in their accumulation. The intensity of the reaction reflects the intensity of inflammation present [7].

In addition to the ARDSnet guidelines which have reduced significantly the complications from mechanical ventilation in ARDS patients [1], the aggressive controlled administration of insulin so as to achieve a borderline hypoglycemic status seems to help improve the gas exchange of these patients as well [8,9]. The goal is to increase the effectiveness of the Na-K ATP depended alveolar membrane ion pump and thus reduce the fluid accumulation in the alveolus. This therapeutic approach has been shown in experimental animal models to increase the gas exchange ratio and improve the acid base balance. Its drawback is the necessity for careful and continuous monitoring of blood glucose levels by the ICU staff.

In conclusions, the JHR is extremely rare and usually associated with the antibiotic treatment of certain bacterial infections but extremely rarely is presented during the course of treatment for coxiella burnetii. Any deterioration of a patient treated in the ICU should not de facto be considered as a new septic episode and adequate time should be allowed for the antibiotic regiments, especially if the culture sets fail to yield new microbes.

## Consent

Written informed consent was obtained from the patient for publication of this case report and accompanying images. A copy of the written consent is available for review by the Editor-in-Chief of this journal.

## Competing interests

The authors declare that they have no competing interests.

## Authors' contributions

"PS and KO analyzed and interpreted the patient data. SA was the responsible doctor of the patient. SG was a major contributor in writing the manuscript. All authors read and approved the final manuscript."
